# Radiologic assessment of a self-shield with boron-containing water for a compact medical cyclotron

**DOI:** 10.1007/s12194-012-0147-4

**Published:** 2012-02-28

**Authors:** Genki Horitsugi, Toshioh Fujibuchi, Ichiro Yamaguchi, Akihisa Eto, Yasuo Iwamoto, Hiromi Hashimoto, Seiki Hamada, Satoshi Obara, Hiroshi Watanabe, Jun Hatazawa

**Affiliations:** 1 MI Clinic, Shoji 1-12-13, Toyonaka, Osaka 560-0004 Japan; 2 Department of Radiological Sciences, School of Health Sciences, Ibaraki Prefectural University of Health Sciences, Ami 4669-2, Ami-machi, Inashiki-gun, Ibaraki, 300-0394 Japan; 3 Department of Environmental Health, National Institute of Public Health, Minami 2-3-6, Wako, Saitama 351-0197 Japan; 4 Yokohama Rousai Hospital, Kodzukue-cho 3211, Kofuku-ku, Yokohama, Kanagawa 222-0036 Japan; 5 Department of Nuclear Medicine and Tracer Kinetics, Osaka University Graduate School of Medicine, Yamada Oka 2-2, Suita, Osaka 565-0871 Japan

**Keywords:** Cyclotron, Boron, Self-shield, Positron emission tomography, Activation, Neutron

## Abstract

The cyclotron at our hospital has a self-shield of boron-containing water. The amount of induced radioactivity in the boron-containing water shield of a compact medical cyclotron has not yet been reported. In this study, we measured the photon and neutron dose rates outside the self-shield during cyclotron operation. We estimated the induced radioactivities of the boron-containing water used for the self-shield and then measured them. We estimated the activation of concrete outside the self-shield in the cyclotron laboratory. The thermal neutron flux during cyclotron operation was estimated to be 4.72 × 10^2^ cm^−2^ s^−1^, and the activation of concrete in a cyclotron laboratory was about three orders of magnitude lower than the clearance level of RS-G-1.7 (IAEA). The activity concentration of the boron-containing water did not exceed the concentration limit for radioactive isotopes in drainage in Japan and the exemption level for Basic Safety Standards. Consequently, the boron-containing water is treatable as non-radioactive waste. Neutrons were effectively shielded by the self-shield during cyclotron operation.

## Introduction

The number of facilities with cyclotrons for producing radionuclides has been increasing as the use of positron emission tomography (PET) has increased [1]. ^18^F-Fluorodeoxyglucose (^18^FDG) is the radionuclide produced in the largest quantities. ^18^FDG can be synthesized from ^18^F, which is produced in a cyclotron by the ^18^O(*p*, *n*)^18^F reaction. Neutrons are generated as a by-product during the production of ^18^F. Thermal neutrons produce radioactive nuclides mainly through the (*n*, γ) reaction in the concrete of the wall, the floor, and the ceiling in the cyclotron laboratory [2, 3]. The amount of neutron flux can be reduced by installation of a self-shield. The thermal neutron flux outside a cyclotron with a self-shield was about 10^2^ − 10^3^ cm^−2^ s^−1^, whereas that without a self-shield was about 10^5^ − 10^7^ cm^−2^ s^−1^ [4–10]. For a cyclotron without a self-shield, the activation of the wall, the floor, the ceiling, and others exceeds the clearance level (CL) of RS-G-1.7 (IAEA) [11]. Thus, the neutron flux can be reduced by three to four orders of magnitude with a self-shield. For a cyclotron with a self-shield, the activation levels outside the self-shield are low except for the floor on which the cyclotron is installed [12].

The compact cyclotron (PETtrace, GE Healthcare, Milwaukee, WI, USA) at our hospital has a self-shield of boron-containing water. Boric acid is used as a neutron-absorbing material in the primary cooling water of a pressurized water reactor in nuclear power plants. There are only two cyclotrons in Japan that have self-shields with boron-containing water, and no experimental studies have been reported for the shielding ability of PETtrace and the amount of induced radioactivity in a self-shield with boron-containing water. It is very important to determine the neutron-shielding ability, and to estimate the activation level in cyclotron laboratories for radiation safety management and for decommissioning of cyclotron facilities.

In this study, we measured the photon and neutron dose rates during cyclotron operation to determine the leakage dose rate outside the self-shield. We also compared the dose rates with the results obtained when the cyclotron was installed, and we confirmed the time-dependent changes in the radiation-shielding ability. We estimated the activation of concrete in the cyclotron laboratory from the obtained neutron dose rate. We estimated the induced radioactivities of the boron-containing water used for the self-shield and then measured them.

## Methods

### Cyclotron performance

We used a PETtrace cyclotron with a self-shield to accelerate negative hydrogen ions (H^−^) and produce ^18^F. This cyclotron was operated almost every weekday. The operating time was 70 min per day with an average beam current of 40 μA and a proton acceleration energy of 16.5 MeV. The self-shield consisted of an approximately 1-m-thick tank filled with boron-containing water (28 m^3^ of water containing 3.5% boron), lead, and polyethylene (including 3% boron as concentration by weight).

### Evaluation of dose rate outside self-shield during cyclotron operation, and time-dependent changes in radiation-shielding ability

**Fig. 1:**
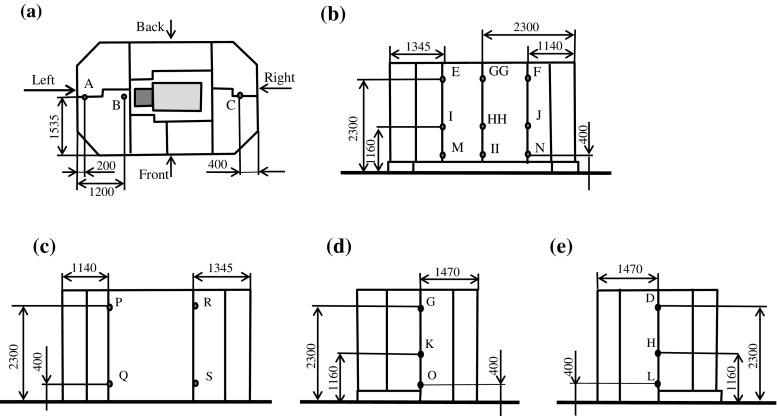
Layout of the cyclotron laboratory showing measurement points (A-II) in the radiation survey. Units of length in figure are mm. **a** Top view, **b** front view, **c** back view, **d** right view, **e** left view

During cyclotron operation, we measured the 1-cm-deep photon dose-equivalent rate using an ionization chamber survey meter (ICS-321B, Aloka, Tokyo, Japan) and the neutron dose-equivalent rate using a rem counter (TPS-451BS, Aloka, Tokyo, Japan) outside the self-shield at 22 points. Figure 1a–e show the measurement points. We performed these measurements in March, 2010. We adopted the maximum measured value at each measurement point as the measured leakage dose rate for the estimation of residual radioactivity in the concrete regarding the safety aspects.

The data obtained immediately after the cyclotron had commenced operation in October 2005 are based on the measurements by the manufacturer. An ionization chamber survey meter (RAM DA-2000+IC-10A-P, ROTEM, Beer-Sheva, Israel) and neutron survey meter (NEUTRON MONITOR 2222A, Studsvik, Nykoping, Sweden) were used for measurement in 2005. These measurements were performed by taking 1 min at each point under the same operational condition of the cyclotron as above. Each dose rate measurement outside the self-shield was carried out under the same operational conditions such as an average beam current of 40 μA, a proton acceleration energy of 16.5 MeV, and exactly at same geometry by use of marked measuring positions. NEUTRON MONITOR 2222A was calibrated by use of a standard Am–Be source in accordance with the recommendations in ICRP Publ. 60, i.e., with a conversion factor of 2.15 μSv/cm^2^ s for Am–Be sources. TPS-451BS was calibrated according to the protocol JIS Z 4521 [13]. Minimum limits of detection were 1 μSv/h in 2005 and 0.1 μSv/h in 2010. We used a *t* test to compare the dose rates measured in the present study with those measured in 2005.

### Estimation of the activation of the concrete walls of cyclotron laboratory

We estimated the residual radioactivity in the concrete walls of the cyclotron laboratory based on the leakage dose rate of neutrons outside the self-shield by assuming 30-year operation under the conditions of 2 h/day and 7 days/week operation. We assumed the 30-year operation for estimation of the residual activity in the concrete concerning the safety aspects, since because a cyclotron is usually operated for about 10–20 years. We estimated the radioactivity of ^60^Co, ^134^Cs, ^152^Eu, and ^154^Eu as the resulting long-lived radionuclides [14–16]. These radionuclides with a long half-life and high cross section are important in decommissioning. Cobalt, cesium, and europium had concentrations of 9.8, 1.3, and 0.55 ppm in concrete, respectively [17, 18]. ^59^Co, ^133^Cs, ^151^Eu, and ^153^Eu have isotopic abundances of 100, 100, 47.81, and 52.19%, respectively [19].

The number of atoms *N* (g^−1^) was calculated as

1 $$ N = \frac{{CN_{A} \theta m}}{M}, $$

where *C* (g^−1^) is the concentration of the target atom in concrete, *N*_*A*_ (mol^−1^) is Avogadro’s number, θ (%) is the isotopic abundance of the target nuclide, *M* (g/mol) is the atomic weight of the target nuclide, and *m* (g) is the mass of the target nuclide.

The thermal neutron flux φ (cm^−2^ s^−1^) was calculated as

2 $$ \varphi = \frac{{Xt_{ 1} }}{{Cft_{ 2} }}, $$

where *X* (μSv/h) is the maximum leakage dose rate of neutrons as measured by a rem counter, *Cf* (=1.06 × 10^−5^ μSv cm^2^) is the neutron fluence-to-dose equivalent conversion coefficient for thermal neutrons given by ICRP Publication 74 [20], *t*_1_ (h) is the operating period per day, and *t*_2_ = 8.64 × 10^4^ s (=24 h × 60 min × 60 s).

The saturation coefficient *S* was calculated as:

3 $$ S = 1 - {\text{exp(}} - \lambda t_{ 3} ) $$

4 $$ \lambda = \frac{0.693}{{T_{ 1 / 2} }}, $$

where λ is the decay constant, *t*_3_ (s) is the operating period, and *T*_1/2_ (s) is the half-life of the target nuclide. ^60^Co, ^134^Cs, ^152^Eu, and ^154^Eu have half-lives of 5.27, 2.07, 13.52, and 8.60 years, respectively [19].

The residual activity concentration *A* (Bq/g) was calculated as

5 $$ A = N\varphi \sigma S . $$

^60^Co, ^134^Cs, ^152^Eu, and ^154^Eu have thermal neutron capture cross sections σ of 37.18 ± 0.06, 29.0 ± 1.5, 5900 ± 200, and 312 ± 7 barns [19].

### Estimation of the activation of the boron-containing water used for the self-shield

We estimated the activity concentration of the boron-containing water used for the self-shield. When the PETtrace is operated at a beam current of 80 μA by use of dual ports, neutrons are generated at a rate of 7.13 × 10^11^ s^−1^ according to the GE Healthcare report [21, 22], and the thermal neutron flux was calculated to be 2.03 × 10^7^ cm^−2^ s^−1^ according to Patterson’s formula [23],

6 $$ \varphi = K\frac{Q}{I}, $$

where *K* (=1.25) is a constant, *Q* (s^−1^) is the total amount of generated neutrons when the cyclotron operates at a beam current of 80 μA with use of dual ports, and *I* (=4.4 × 10^4^ cm^2^) is the total interior surface area of the self-shield.

The activity concentration of the boron-containing water for the thermal neutron capture reaction was calculated from the thermal neutron flux. The concentrations of the principal heavy metals in the boron-containing water were based on water quality test results for tap water in 2006 for Toyonaka City, Osaka, Japan (the location of the hospital) given in the Database of Water Quality of Aqueduct [24]. The cobalt concentration was taken to be 10 μg/L [25–28]. The isotopic abundances and the thermal neutron capture reaction cross sections of the radioactive nuclides were obtained from the 11th edition of Radioisotope Pocket Data [19]. The cyclotron operation period was taken to be 30 years (the same value used in the evaluation of the activation of concrete), and the radioactive nuclide concentrations 1 week after operation of the cyclotron were calculated. The ratios of the radioactive nuclide concentrations to the concentration limits for drainage in Japan and the exemption level for Basic Safety Standards (BSS) [29] were also calculated.

### Measurement of activation of boron-containing water

**Fig. 2:**
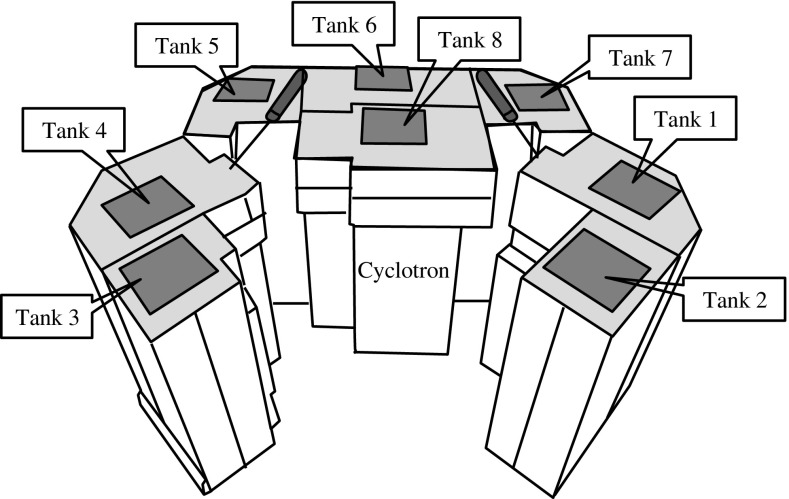
Layout of the water tanks for the self-shield

Figure 2 shows the layout of the self-shield. It consists of eight tanks positioned about the target. The boron-containing water analyzed in the present study was from tank 3, which was near the target. Gamma-ray spectra of a 100 cm^3^ sample were obtained with use of a high-purity germanium semiconductor detector (GMX-20195-S, CFG-LB-GMX-SV, ORTEC, Oak Ridge, Tennessee, USA) over a time of 50 min, and the measurement range was from 22–1638 keV. The high-purity germanium semiconductor detector was calibrated with mixed radionuclide gamma-ray reference solution (Mixed nuclide standardized solution, Japan Radioisotope Association, Tokyo, Japan), which consists of ^109^Cd (88.03 keV), ^57^Co (122.1 keV), ^139^Ce (165.9 keV), ^203^Hg (279.2 keV), ^113^Sn (391.7 keV), ^85^Sr (514.9 keV), ^137^Cs (661.6 keV), ^88^Y (898.0 and 1836 keV) and ^60^Co (1173 and 1333 keV), and with a standard radionuclide gamma source (Radioactivity standard solution, Japan Radioisotope Association, Tokyo, Japan) that consists of ^125^I (27 and 35 keV). The detector sensitivities for targeted gamma rays were 0.817% (^60^Co), 0.712% (^24^Na) and 0.855% (^65^Zn), respectively. The limit of detection was set to 3σ (standard deviation) of the background count as defined in the International Union of Pure and Applied Chemistry (IUPAC). The preset time for gamma-ray spectral analysis was 50 min, since each measurement would be able to detect the activity over the concentration limit for drainage. Targeted gamma rays of ^60^Co, ^24^Na, and ^65^Zn were set to be 1173.24, 1368.6, and 1115.52 keV. These energy window settings and the energy resolution (FWHM) of the detector were 1170.5–1176.1 keV (1.77 keV), 1365.7–1371.6 keV (1.91 keV), and 1112.5–1118.5 keV (1.72 keV), respectively. Nuclides whose gamma-ray energies lie outside the measurement range, as well as beta emitters, were excluded.

The ^3^H concentration of a 5.0 cm^3^ sample that had been distilled prior to the measurement was determined with a liquid scintillation counter (Tri-Carb 2900TR, Perkin Elmer, Massachusetts, USA) over 40 min.

## Results

### Evaluation of dose rate outside the self-shield during cyclotron operation, and time-dependent changes in radiation-shielding ability

Table 1 lists the 1-cm-deep dose-equivalent rate at each measurement point. The neutron dose rate at point P could not be measured because of the arrangement of peripheral equipment. The neutron and photon dose rates had maximum values at the target. The second-highest neutron dose rate was measured near the floor in the vicinity of the target, whereas the second-highest photon dose rate was near the opening and closing sections of the self-shield. The average neutron and photon dose rates were 3.1 μSv/h (range from 0.2 to 18.0) and 17.4 μSv/h (range from 2.4 to 50.1), respectively.

**Table 1:** Photon and neutron dose rates outside the self-shield by survey meter in 2010

Point	Photon (μSv/h)	Neutron (μSv/h)
A	9.7	2.5
B	50.1	18.0
C	5.7	0.6
D	5.9	1.5
E	10.9	0.6
F	14.1	0.5
G	2.4	0.2
H	12.1	4.0
I	14.4	3.0
J	14.1	0.5
K	6.4	3.0
L	15.8	7.0
M	22.3	10.0
N	22.3	0.8
O	10.1	0.4
P	14.4	No data
Q	11.5	0.3
R	8.7	0.2
S	12.4	1.8
GG	25.9	0.6
HH	45.3	1.3
II	48.3	9.0

Measurement points are shown in Fig. 1. The photon dose rate was measured by an ionization chamber survey meter (ICS-321B), and the neutron dose rate was measured by a rem counter (TPS-451BS)

**Fig. 3:**
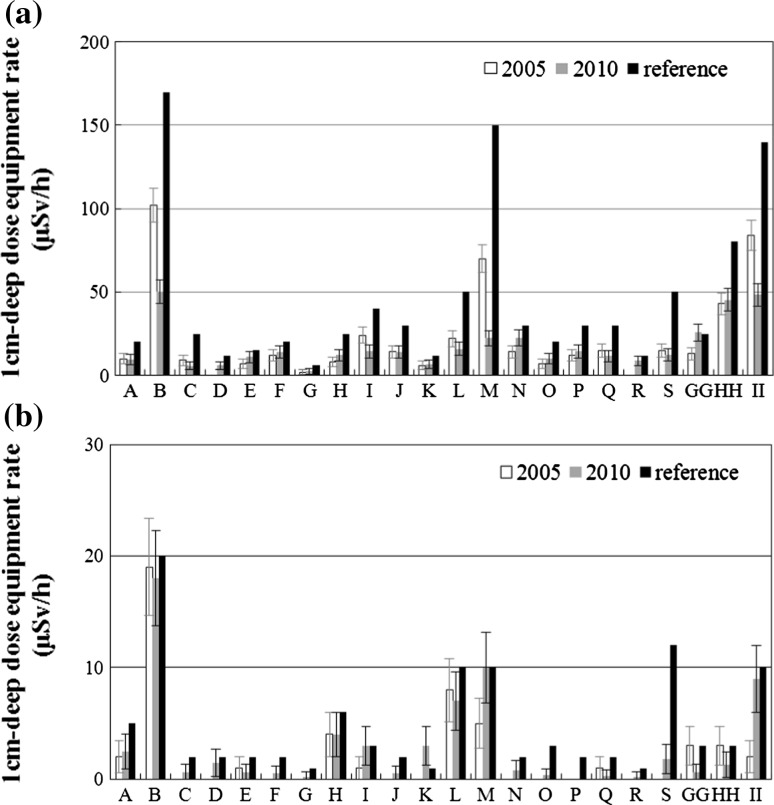
Time-dependent changes in leakage dose rates during cyclotron operation for **a** photons and **b** neutrons. *Error bars* show ±SD. The cyclotron was operated with beam current of 40 μA, a proton acceleration energy of 16.5 MeV, and same port in 2005 and in 2010. Measurement points (A-II) correspond to points in Fig. 1

Figures 3a and b show the photon and neutron dose rates measured in 2005 and 2010 compared with reference values given by the cyclotron manufacturer. The average leakage dose rate outside the self-shield did not vary by more than 1σ of the dose distribution at each measurement point. No significant difference was observed over the 5-year period (*p* = 0.05).

### Estimation of the activation of the concrete walls of the cyclotron laboratory

Table 2 lists the residual radioactivity of the concrete walls after the cyclotron had been operated for 30 years. The thermal neutron flux during cyclotron operation was estimated to be 4.72 × 10^2^ cm^−2^ s^−1^ as a result of calculation. ^152^Eu was estimated to have the highest ratio to the CL (*D*/*C*, where *D* is the specific activity of a radionuclide in the component and *C* is the CL of the radionuclide) (1.9 × 10^−3^ ± 1.8 × 10^−3^). The sum of the ratio to the CL (Σ*D*/*C*) was 3.5 × 10^−3^ ± 2.5 × 10^−3^. Assuming that the thermal neutron flux during operation is 1.38 × 10^5^ cm^−2^ s^−1^, the Σ*D*/*C* for the concrete in the cyclotron laboratory is estimated to be almost 1.

**Table 2:** Activation of concrete walls of the cyclotron laboratory and comparison with the CL (RS-G-1.7)

Target element	Radionuclide	Concentration of natural atom (ppm)	Abundance (%)	Cross section (barn)	Half-life (year)	Activity concentration (Bq/g)	CL (Bq/g)	*D*/*C*
						Mean ± SD		Mean ± SD
Cobalt	^60^Co	9.8 ± 10.3	100	37.18 ± 0.06	5.27	1.4 × 10^−4^ ± 1.5 × 10^−4^	0.1	1.4 × 10^−3^ ± 1.5 × 10^−3^
Cesium	^134^Cs	1.3 ± 1.8	100	29.0 ± 1.5	2.07	6.7 × 10^−6^ ± 9.4 × 10^−6^	0.1	6.7 × 10^−5^ ± 9.4 × 10^−5^
Europium	^152^Eu	0.55 ± 0.38	47.81	5900 ± 200	13.54	1.9 × 10^−4^ ± 1.4 × 10^−4^	0.1	1.9 × 10^−3^ ± 1.4 × 10^−3^
	^154^Eu	0.55 ± 0.38	52.19	312 ± 7	8.60	1.3 × 10^−5^ ± 9.0 × 10^−6^	0.1	1.3 × 10^−4^ ± 9.0 × 10^−5^
							Σ*D*/*C*	3.5 × 10^−3^ ± 2.0 × 10^−3^

### Estimation of the activation of boron-containing water used as a self-shield

Table 3 lists the concentrations of the activation nuclides, the ratio to the concentration limit for radioactive isotopes in drainage, and the ratio to the BSS exemption levels. ^60^Co had the highest ratio to the concentration limit for drainage (2.2 × 10^−2^ ± 3.5 × 10^−3^). The sum of the ratios to the concentration limit for drainage was 1.6 × 10^−1^ ± 1.8 × 10^−2^. ^60^Co had the highest ratio to the BSS exemption level (2.2 × 10^−3^ ± 3.5 × 10^−4^). The sum of the ratios to the BSS exemption level was 2.4 × 10^−3^ ± 3.5 × 10^−4^.

**Table 3:** Estimated activation of the boron-containing water used in the self-shield

Target element	Radionuclide	Concentration of natural atom (μg/l)	Abundance (%)	Cross section (barn)	Half-life	Activity concentration (Bq/g)	Concentration limit for drainage (Bq/cc)	Ratio to concentration limit for drainage	Exemption level (Bq/g)	Ratio to BSS exemption level
						Mean ± SD		Mean ± SD		Mean ± SD
Chlorine	^36^Cl	2.35 × 10^4^	75.77	43.6 ± 0.4	3.01 × 10^5^ years	5.4 × 10^−3^ ± 8.6 × 10^−4^	0.9	6.0 × 10^−3^ ± 9.6 × 10^−4^	1.0 × 10^4^	5.4 × 10^−7^ ± 8.6 × 10^−8^
	^38^Cl	2.35 × 10^4^	24.22	0.433 ± 0.006	0.62 h	7.7 × 10^−83^ ± 1.2 × 10^−83^	7	1.1 × 10^−83^ ± 1.8 × 10^−84^	1.0 × 10^1^	7.7 × 10^−84^ ± 1.2 × 10^−84^
Iron	^55^Fe	3.0 × 10^1^	5.8	2.25 ± 0.18	2.73 years	2.5 × 10^−4^ ± 4.5 × 10^−5^	2	1.3 × 10^−4^ ± 2.3 × 10^−5^	1.0 × 10^4^	2.5 × 10^−8^ ± 4.5 × 10^−9^
	^59^Fe	3.0 × 10^1^	2.8	1.28 ± 0.05	44.5 days	5.8 × 10^−6^ ± 9.6 × 10^−7^	0.4	1.5 × 10^−5^ ± 2.4 × 10^−6^	1.0 × 10^1^	5.8 × 10^−7^ ± 9.6 × 10^−8^
Manganese	^56^Mn	5.0 × 10^0^	100	13.3 ± 0.2	2.58 h	1.0 × 10^−22^ ± 1.7 × 10^−23^	3	3.5 × 10^−23^ ± 5.6 × 10^−24^	1.0 × 10^1^	1.0 × 10^−23^ ± 1.7 × 10^−24^
Cobalt	^60^Co	1.0 × 10^1^	100	37.18 ± 0.06	5.27 years	2.2 × 10^−2^ ± 3.5 × 10^−3^	0.2	1.1 × 10^−1^ ± 1.7 × 10^−2^	1.0 × 10^1^	2.2 × 10^−3^ ± 3.5 × 10^−4^
Copper	^64^Cu	1.0 × 10^2^	69.17	4.50 ± 0.02	12.7 h	1.8 × 10^−6^ ± 2.9 × 10^−7^	7	2.6 × 10^−7^ ± 4.1 × 10^−8^	1.0 × 10^2^	1.8 × 10^−8^ ± 2.9 × 10^−9^
	^66^Cu	1.0 × 10^2^	30.83	2.17 ± 0.03	0.09 h	0	5	0		
Zinc	^65^Zn	1.0 × 10^2^	48.63	0.76 ± 0.02	244.3 days	2.0 × 10^−3^ ± 3.2 × 10^−4^	0.2	9.9 × 10^−3^ ± 1.6 × 10^−3^	1.0 × 10^1^	2.0 × 10^−4^ ± 3.2 × 10^−5^
	^69^Zn	1.0 × 10^2^	18.75	1.0 ± 0.1	0.94 h	1.5 × 10^−57^ ± 2.9 × 10^−58^	30	5.1 × 10^−59^ ± 9.6 × 10^−60^	1.0 × 10^4^	1.5 × 10^−61^ ± 2.9 × 10^−62^
Hydrogen	^3^H	1.1 × 10^8^	0.0115	0.000519 ± 0.000007	12.32 years	1.9 × 10^−2^ ± 3.1 × 10^−3^	20	9.5 × 10^−4^ ± 1.5 × 10^−4^	1.0 × 10^6^	1.9 × 10^−8^ ± 3.1 × 10^−9^
Cadmium	^107^Cd	1.0 × 10^0^	1.25	0.97	6.5 h	6.7 × 10^−15^ ± 1.1 × 10^−15^	10	6.7 × 10^−16^ ± 1.1 × 10^−16^		
	^109^Cd	1.0 × 10^0^	0.89	1.1 ± 0.3	461.4 days	3.2 × 10^−7^ ± 1.0 × 10^−7^	0.4	7.9 × 10^−7^ ± 2.5 × 10^−7^	1.0 × 10^2^	3.2 × 10^−10^ ± 1.0 × 10^−10^
	^113m^Cd	1.0 × 10^0^	24.13	2.2 ± 0.5	14.1 years	1.3 × 10^−5^ ± 3.6 × 10^−6^	0.04	3.2 × 10^−4^ ± 8.9 × 10^−5^	1.0 × 10^3^	1.3 × 10^−8^ ± 3.6 × 10^−9^
	^115^Cd	1.0 × 10^0^	28.73	0.30 ± 0.02	53.46 h	3.0 × 10^−7^ ± 5.2 × 10^−8^	60	5.0 × 10^−9^ ± 8.7 × 10^−10^	1.0 × 10^2^	3.0 × 10^−9^ ± 5.2 × 10^−10^
	^117^Cd	1.0 × 10^0^	7.49	0.050 ± 0.008	2.49 h	5.6 × 10^−28^ ± 1.3 × 10^−28^	3	1.9 × 10^−28^ ± 4.2 × 10^−29^		
Mercury	^197^Hg	5.0 × 10^−2^	0.15	3080 ± 180	64.94 h	6.9 × 10^−7^ ± 1.2 × 10^−7^	4	1.7 × 10^−7^ ± 2.9 × 10^−8^	1.0 × 10^2^	6.9 × 10^−9^ ± 1.2 × 10^−9^
	^203^Hg	5.0 × 10^−2^	29.86	4.89 ± 0.05	46.61 days	1.1 × 10^−6^ ± 1.8 × 10^−7^	2	5.7 × 10^−7^ ± 9.2 × 10^−8^	1.0 × 10^2^	1.1 × 10^−8^ ± 1.8 × 10^−9^
Selenium	^75^Se	1.0 × 10^0^	0.89	51.8 ± 1.2	119.8 days	2.1 × 10^−5^ ± 3.4 × 10^−6^	0.3	7.0 × 10^−5^ ± 1.1 × 10^−5^	1.0 × 10^2^	2.1 × 10^−7^ ± 3.4 × 10^−8^
Lead	^209^Pb	1.0 × 10^0^	52.4	0.00049 ± 0.00003	3.25 h	1.2 × 10^−24^ ± 2.0 × 10^−25^	10	1.2 × 10^−25^ ± 2.0 × 10^−26^		
Arsenic	^76^As	1.0 × 10^0^	100	4.5 ± 0.1	25.87 h	2.3 × 10^−6^ ± 3.8 × 10^−7^	0.5	4.7 × 10^−6^ ± 7.6 × 10^−7^	1.0 × 10^2^	2.3 × 10^−8^ ± 3.8 × 10^−9^
Aluminum	^28^Al	4.0 × 10^1^	100	0.231 ± 0.003	0.04 h	0	80	0		
Sodium	^24^Na	2.14 × 10^4^	100	0.530 ± 0.005	14.96 h	7.2 × 10^−4^ ± 1.2 × 10^−4^	2	3.6 × 10^−4^ ± 5.8 × 10^−5^	1.0 × 10^1^	7.2 × 10^−5^ ± 1.2 × 10^−5^
Calcium	^41^Ca	2.04 × 10^4^	96.94	0.41 ± 0.02	1.02 × 10^5^ years	1.4 × 10^−4^ ± 2.4 × 10^−5^	4	2.9 × 10^−5^ ± 6.0 × 10^−6^		
	^45^Ca	2.04 × 10^4^	2.09	0.88 ± 0.05	162.7 days	2.9 × 10^−2^ ± 4.9 × 10^−3^	1	2.9 × 10^−2^ ± 4.9 × 10^−3^	1.0 × 10^4^	2.9 × 10^−6^ ± 4.9 × 10^−7^
	^47^Ca	2.04 × 10^4^	0.004	0.74 ± 0.07	4.54 days	1.6 × 10^−5^ ± 2.9 × 10^−6^	0.5	3.2 × 10^−5^ ± 5.9 × 10^−6^	1.0 × 10^1^	1.6 × 10^−6^ ± 2.9 × 10^−7^
Magnesium	^27^Mg	1.24 × 10^4^	11.01	0.0382 ± 0.0008	0.16 h	0	40	0		
Total								1.6 × 10^−1^ ± 1.8 × 10^−2^		2.4 × 10^−3^ ± 3.5 × 10^−4^

### Measurement of activation of boron-containing water

**Fig. 4:**
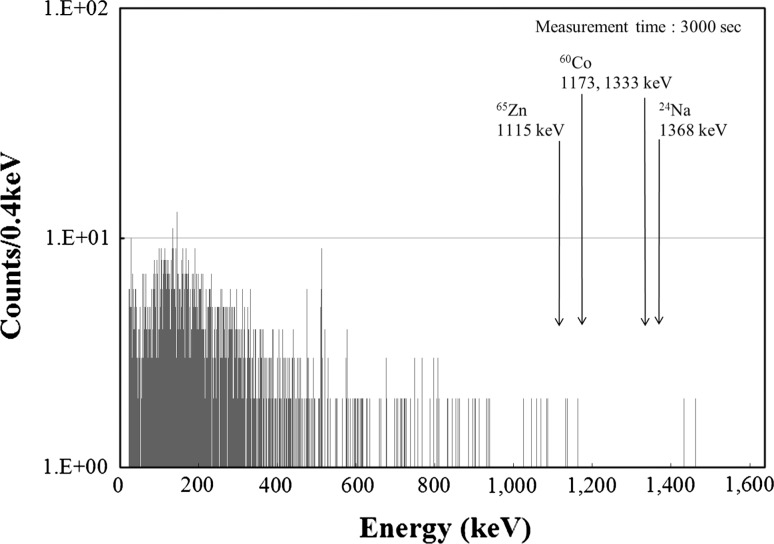
Gamma-ray spectra obtained from the boron-containing water

Table 4 shows the results for the measured activation of the boron-containing water, and Fig. 4 shows a gamma-ray spectrum measured for the boron-containing water. No significant peaks for assumed nuclides were observed in the gamma-ray spectra. Thus, the concentrations for all activated nuclides were below the detection limit. ^3^H also had a concentration below the detection limit.

**Table 4:** Evaluated activation of the boron-containing water and comparison with concentration limits for drainage and BSS exemption level

Target element	Radionuclide	Detection limit (Bq/cm^3^)	Concentration limit for drainage (Bq/cc)	Ratio to concentration limit for drainage	Exemption level (Bq/g)	Ratio to BSS exemption level
Hydrogen	^3^H	1.7 × 10^−2^	60	<2.8 × 10^−4^	1 × 10^6^	<1.7 × 10^−8^
Cobalt	^60^Co	5.7 × 10^−3^	0.2	<2.9 × 10^−2^	10	<5.7 × 10^−4^
Zinc	^65^Zn	9.2 × 10^−3^	0.2	<4.6 × 10^−2^	10	<9.2 × 10^−4^
Sodium	^24^Na	4.7 × 10^−3^	2	<2.4 × 10^−3^	10	<4.7 × 10^−4^
Total				<7.7 × 10^−2^		<2.0 × 10^−3^

To evaluate the safety aspects, when we considered that all nuclides had concentrations equal to the detection limit, the sum of the ratios to the concentration limit for drainage was estimated to be 7.7 × 10^−2^, and the sum of the ratios to the BSS exemption level was 2.0 × 10^−3^. This confirms that the concentrations were sufficiently below the concentration limits for drainage and the BSS exemption levels.

## Discussion

When the cyclotron was operated 2 h/day and 7 days/week, the average leakage dose rate during operation was 0.287 mSv/week, which is equivalent to about a 25% effective dose limit of 1.0 mSv/week for places in which registered radiation workers can freely enter any time into radiation-controlled areas. Neutron dose rates at 1 m from the surface of the self-shield of an 11 MeV cyclotron that is shielded with mainly the boron-containing concrete and operated with a beam current of 40 μA were reported to be 0.03–14.2 μSv/h (the average was 6.6 μSv/h) by Pant et al. [30], which is consistent with this study within 1 order of magnitude. Even if the neutron dose rate was 10 times higher than this result, the activation in the concrete is not expected to exceed the CL. Thus, these data show that the self-shields effectively shielded neutrons during cyclotron operation.

These measurements were carried out by use of two survey meters. These were calibrated according to the standard method, and the response characteristics of these two survey meters were almost the same. The average leakage dose rate outside the self-shield did not vary by more than 1σ of the dose distribution at each measurement point. Therefore, we consider that the time-dependent changes in the radiation-shielding ability were negligible. It is reasonable to consider that time-dependent changes in the radiation-shielding ability are small, because the quantity of boron-containing water can be managed well easily.

From Eq. 2, the thermal neutron flux during cyclotron operation was estimated to be 4.72 × 10^2^ cm^−2^ s^−1^, and the activation of the concrete walls of the cyclotron laboratory was about three orders of magnitude lower than the CL. The thermal neutron flux during cyclotron operation was similar to those reported for other cyclotrons [8–10]. Assuming that the thermal neutron flux during operation is more than 1.38 × 10^5^ cm^−2^ s^−1^, the Σ*D*/*C* for the concrete in the cyclotron laboratory exceeds 1. The activation of the concrete walls, the floor, and the ceiling is sufficiently low so that it is not necessary to treat the concrete as radioactive waste when the cyclotron is shut down. The residual radioactivity in concrete was not measured in this study, because it is not practical to take samples of concrete from the walls and floor.

The estimated activity concentration of the boron-containing water in the self-shield was low enough so that the water could be released into the environment. Moreover, the activity concentration of the boron-containing water did not exceed the BSS exemption level. The neutron flux was overestimated, because all neutrons were regarded as thermal neutrons in our estimates.

We assumed that the concentrations of the heavy metals in the boron-containing water were similar to those in tap water for this calculation. It is thus plausible that the activation of the boron-containing water was estimated adequately because the concentrations of the heavy metals in the boron-containing water seemed to be almost equal to that in tap water. However, the concentrations of the heavy metals in the boron-containing water depend on the characteristics of tap water in each region. In the measurement of the amount of the activity in the boron-containing water, all of the evaluated radionuclides had concentrations below the concentration detection limit. When the concentrations of all evaluated radionuclides were assumed to be at their upper limits of detection, the concentrations were about two orders of magnitude below the concentration limit for drainage and about three orders of magnitude below the BSS exemption level. Even for this overestimate, the activity concentration of the boron-containing water did not exceed the concentration limit for drainage and the BSS exemption level. Consequently, the boron-containing water can be treated as non-radioactive drainage.

As the condition for the boron-containing water is treatable as non-radioactive waste because of its low concentration, we summarized the advantages and disadvantages of boron-containing water used as a neutron shield by comparing it with a solid-type self-shield such as boron-containing concrete. The advantages of boron-containing water are a lower initial cost, a smaller amount of radioactive waste, and the ease of recycling boron at decommission. Disadvantages are boron exposure to engineers during maintenance, and potential problems caused by water handling. Although it is not necessary to treat the boron-containing water as radioactive drainage, it is important to consider various methods for processing it. Boron is designated as a toxic substance by the Water Pollution Control Law and the Soil Contamination Countermeasures Law in Japan. It is thus essential to consider the environment when disposing of boron. It is important to satisfy the uniform national effluent standards in Japan (non-coastal areas: 10 mg/l) and the effluent standards of the specific municipality (for example, in drinking water sources region of Osaka prefecture, 1.0 mg/l is assigned as the limit for drainage.) when releasing the boron-containing water. For example, it is necessary to dilute the boron-containing water with about 1.6 × 10^6^ tons of water to satisfy the effluent standards of Osaka (where the hospital is located). Therefore, recycling of boron should be considered instead of release of boron-containing water. However, because the boron-containing water does not need to be treated as radioactive waste, all of these methods can be used. When boron-containing water is processed, not only is it necessary to consider the cost, but it is also important to obtain the agreement of the public and to consider the environment.

## Conclusion

The activation of the concrete outside the self-shield in a cyclotron laboratory is sufficiently below the CL. It is not necessary to treat the boron-containing water as radioactive waste. Neutrons were effectively shielded by the self-shield during cyclotron operation.
